# A survey of paediatric HIV programmatic and clinical management practices in Asia and sub-Saharan Africa—the International epidemiologic Databases to Evaluate AIDS (IeDEA)

**DOI:** 10.7448/IAS.16.1.17998

**Published:** 2013-01-15

**Authors:** IeDEA Pediatric Working Group

**Keywords:** paediatric HIV, cohort, observational, Africa, Asia

## Abstract

**Introduction:**

There are limited data on paediatric HIV care and treatment programmes in low-resource settings.

**Methods:**

A standardized survey was completed by International epidemiologic Databases to Evaluate AIDS paediatric cohort sites in the regions of Asia-Pacific (AP), Central Africa (CA), East Africa (EA), Southern Africa (SA) and West Africa (WA) to understand operational resource availability and paediatric management practices. Data were collected through January 2010 using a secure, web-based software program (REDCap).

**Results:**

A total of 64,552 children were under care at 63 clinics (AP, *N*=10; CA, *N=*4; EA, *N=*29; SA, *N=*10; WA, *N*=10). Most were in urban settings (*N*=41, 65%) and received funding from governments (*N*=51, 81%), PEPFAR (*N*=34, 54%), and/or the Global Fund (*N*=15, 24%). The majority were combined adult–paediatric clinics (*N*=36, 57%). Prevention of mother-to-child transmission was integrated at 35 (56%) sites; 89% (*N*=56) had access to DNA PCR for infant diagnosis. African (*N*=40/53) but not Asian sites recommended exclusive breastfeeding up until 4–6 months. Regular laboratory monitoring included CD4 (*N*=60, 95%), and viral load (*N*=24, 38%). Although 42 (67%) sites had the ability to conduct acid-fast bacilli (AFB) smears, 23 (37%) sites could conduct AFB cultures and 18 (29%) sites could conduct tuberculosis drug susceptibility testing. Loss to follow-up was defined as >3 months of lost contact for 25 (40%) sites, >6 months for 27 sites (43%) and >12 months for 6 sites (10%). Telephone calls (*N*=52, 83%) and outreach worker home visits to trace children lost to follow-up (*N*=45, 71%) were common.

**Conclusions:**

In general, there was a high level of patient and laboratory monitoring within this multiregional paediatric cohort consortium that will facilitate detailed observational research studies. Practices will continue to be monitored as the WHO/UNAIDS Treatment 2.0 framework is implemented.

## Introduction

UNAIDS estimates that 3.4 million children under the age of 15 years are currently living with HIV infection; 91% of these children reside in sub-Saharan Africa [1]. HIV infection in children progresses rapidly without treatment – over half of the HIV-infected children in Africa die by the age of two years if they do not receive antiretroviral therapy (ART) [2]. Highly active antiretroviral therapy (HAART) results in marked survival benefits for HIV-infected children [3]. However, at the end of 2011, only 28% of the 2 million children in need of treatment were receiving it, compared to 57% in adults; in many countries, the disparity between paediatric and adult access to treatment is even greater [1–4]. There are multiple bottlenecks that limit paediatric treatment, including poor access to early HIV diagnosis in infants, lack of health care centres and providers equipped to deliver paediatric treatment, limited availability of paediatric drug formulations and the high cost of such formulations, the complexity of adjusting dosing as a child ages and weak systems for patient retention.

To date, data are limited with regards to the structure and outcomes of paediatric HIV care and treatment programmes in resource-constrained settings. Understanding the availability of clinical management resources as well as the application of prevention, diagnosis and treatment standards is necessary to provide a context for observational cohort studies. In turn, such studies are necessary to optimize programme development, and to improve patient access and outcomes. While information about local or national programmes may be available, the indicators used are not necessarily comparable between countries or across regions [5–7]. Large data gaps on the numbers of children living with HIV and those who meet criteria for treatment initiation [8] make it even more difficult to project the level of additional resources needed to deliver paediatric HIV care.

The International epidemiologic Databases to Evaluate AIDS (IeDEA; www.iedea.org) is a global network that includes paediatric cohorts located in Asia; the Caribbean and South America; and Central, East, Southern, and West Africa [9,10]. IeDEA provide a unique platform to evaluate the health systems that support prevention, care and treatment in some of the most experienced paediatric HIV cohorts in these regions [11,12]. A global survey was conducted in 2010 to determine and compare the resources and care structures of paediatric ART programmes across the IeDEA regions.

## Methods

### Site assessment tool

Regional investigators developed and standardized a site assessment tool that included questions regarding site resources, clinical practices and access to drugs and laboratory monitoring for adult, paediatric and combined adult–paediatric clinical settings. Sites that had either paediatric-only or combined paediatric–adult programmes were asked to respond to 253 questions regarding general programme resources and clinical management (*N*=110), general cancer diagnostics and management (*N*=44), paediatric-specific information (*N*=61) and paediatric cancer diagnostics and management (*N*=38). Multi-lingual versions of the site assessment tool were implemented using the REDCap (Research Electronic Data Capture) system, a secure, web-based application designed to support data capture for research studies (project-redcap.org). REDCap was developed at the Vanderbilt Institute for Clinical and Translational Research. It provides an interface for validated data entry, audit trails for tracking data manipulation and automated import and export procedures to common statistical packages [13]. Separate REDCap databases were created for each of the participating regions and could be completed directly online or transferred from paper copies.

### Data collection

Regional data centres coordinated distribution to and completion of the survey by the sites. Clinical site investigators affiliated with the IeDEA regional networks were primarily responsible for completing the surveys. Data collection was completed by January 2010. Sites in Southern Africa had completed a comparable regional survey between January and March 2009, and data from that survey were entered into the IeDEA assessment tool, as appropriate. Additional data on antiretroviral drug access from Southern Africa were collected in April 2009. In the event that key data were missing from the Southern African survey, their sites were asked to answer additional questions during the analysis phase. All data were entered and merged into the REDCap system before analysis.

### Analysis

Site survey data were received from the five IeDEA regions that had active paediatric HIV cohort databases: Asia, Central Africa, East Africa, Southern Africa and West Africa. For the purpose of analysis, only individual clinical sites that were actively contributing individual patient data to their regional paediatric cohort at the time of the assessment were included.

Ten data categories were selected *a priori* for analysis, including basic site characteristics, clinical service provision, availability of laboratory services, prevention of mother-to-child transmission (PMTCT) interventions, HIV diagnostic testing, access to and use of antiretroviral drugs, management of tuberculosis (TB), prophylaxis for *Pneumocystis jiroveci* pneumonia, malaria management, and follow-up and transfer practices. Categories were selected based on clinical and programmatic relevance. Site-level responses were evaluated for inconsistencies and sent to the IeDEA regional paediatric representatives to coordinate an additional quality control check, as needed. The corrected individual data summaries were combined to produce a regional-level data summary report.

## Results

### Programme characteristics

Data were collected from all 63 sites contributing data on 64,762 children exposed to and/or living with HIV to the IeDEA cohort for regional and multiregional analyses. This included 10 clinics/sites in Asia, 4 in Central Africa, 29 in East Africa, 10 in Southern Africa and 10 in West Africa (Table 1). The majority were public government-run clinics (73%) in urban settings (65%), providing care in combined adult–paediatric clinics (57%). Clinics received core programme and clinical support funding through multiple sources, including their own local governments (*N*=51, 81%), research grants (*N*=36; 57%), the US PEPFAR programme (*N*=34, 54%), the Global Fund (*N*=15, 24%) and patient fees (*N*=9, 14%). None of the sites required patients to pay for first-line ART; two sites in West Africa required partial payment for second-line ART.

**Table 1:** Programme characteristics of participating clinical centres*

	Asia	Central Africa	East Africa	Southern Africa	West Africa

	*N*=10	*N*=4	*N*=29	*N*=10	*N*=10
Children under care, *N*	1416	475	29,772	30,864	2025
Public/government clinic	6 (60)	1 (25)	24 (83)	8 (80)	7 (70)
Clinic setting					
- Urban	8 (80)	3 (75)	12 (41)	10 (100)	8 (80)
- Mixed urban–rural	2 (20)	0	14 (48)	0	1 (10)
Sources of funding					
- Government	10 (100)	3 (75)	24 (83)	9 (90)	5 (50)
- Research grants	4 (40)	0	18 (62)	9 (90)	5 (50)
- US PEPFAR	0	0	25 (86)	7 (70)	2 (20)
- Global Fund	4 (40)	2 (50)	1 (3)	4 (40)	4 (40)
- Patient fees	1 (10)	2 (50)	1 (3)	2 (20)	3 (30)
Mixed adult–paediatric patient cohort	0	3 (75)	27 (93)	5 (50)	1 (10)
Upper age limit, median years (IQR)	18 (15–18)	15 (14.8–15.5)	14 (13–14)	18 (16–18)	15 (15–16)
Half-day dedicated paediatric clinic sessions, median sessions (IQR)	1.5 (1–2.8)	2.5 (0.8–4.3)	1 (1–4)	10 (10–10)	5 (3.5–5)
PMTCT embedded into clinical care at site	4 (40)	1 (25)	25 (86)	2 (20)	3 (30)
Most commonly recommended infant feeding approach exclusive breastfeeding until 4–6 months	0	2 (50)	29 (100)	4 (40)	5 (50)
Food supplements for malnourished children	3 (30)	2 (50)	27 (93)	8 (80)	5 (50)
Presence of support group for children	6 (60)	2 (50)	12 (41)	8 (80)	3 (30)
Disclosure counselling available for caregivers	8 (80)	1 (25)	28 (97)	9 (90)	5 (50)
Disclosure counselling available for children	7 (70)	2 (50)	24 (83)	8 (80)	4 (40)
Interventions following missed visit					
- Phone call	9 (90)	3 (75)	25 (86)	9 (90)	6 (60)
- Home visit by clinic staff	4 (40)	1 (25)	5 (17)	0	3 (30)
- Home visit by outreach worker	5 (50)	4 (100)	26 (90)	7 (70)	3 (30)
Definition of lost to follow-up: duration of absence					
- >3 months	0	2 (50)	10 (34)	7 (70)	6 (60)
- >6 months	3 (30)	2 (50)	19 (66)	1 (10)	2 (20)
- >12 months	5 (50)	0	0	0	1 (10)

IQR, interquartile range; PMTCT, prevention of mother-to-child HIV transmission programme.

* Data listed as *N* (%) unless otherwise specified.

The median upper age limit of children attending the clinic ranged from 14 years at participating sites in East Africa to 18 years in Asia and Southern Africa. The median number of half-day clinic sessions per week dedicated to children ranged from one in East Africa to 10 in Southern African sites; paediatric care provided during mixed adult–paediatric clinics is not reflected in this response. Direct food assistance was uncommon in Asia (30%) but available for malnourished children in almost all East African sites (93%). Across the regions, half of the sites had support groups for children, and most offered disclosure counselling for caregivers (81%) and children (71%).

After a missed clinic visit, sites utilized phone calls (83%), home visits by clinic staff (21%) and home visits by outreach workers (71%) to track patients and reschedule appointments, when feasible. Loss to follow-up was defined as >3 months of lost contact for 25 (40%) sites, >6 months for 27 sites (43%) and >12 months for 6 sites (10%). The reasons reported by sites for patient loss included non-disclosure to family or neighbours (19%), death (35%) and lack of financial resources within the family to sustain regular care (44%).

Outside of East Africa, PMTCT programmes were less commonly embedded into clinical care. Maternal antiretroviral regimens varied among sites and ranged from single-dose nevirapine at delivery to triple-drug ART. Infant care after delivery most commonly included post-natal antiretrovirals (79%), early infant diagnostic testing (79%) and co-trimoxazole prophylaxis until infection status was confirmed (81%). Sites reported their most commonly recommended infant feeding approaches, with 63% preferring exclusive breastfeeding through to 4–6 months of age, and 21% preferring formula/replacement feeding at the time of the survey.

### Co-infection prophylaxis and management

PCP prophylaxis was not discontinued in children on therapy at some sites within the African regions (e.g., 86% in East Africa, 20% in West and Southern Africa), while all Asian sites stopped co-trimoxazole after immunologic improvement on ART. With regards to malaria, most (70%) African sites reported that they used blood smears for diagnosis. Available diagnostic testing for paediatric TB included skin testing (33%), chest x-ray (89%), acid-fast bacilli smear (67%) and culture (37%), as well as gastric washing (35%) and induced sputum (21%) on-site. Most reported that TB treatment was available (79%), with children at 60% of these sites being offered directly observed therapy. Almost all sites reported that isoniazid (92%), rifampicin (92%) and pyrazinamide (89%) were standard drugs in first-line induction therapy for TB in children, while 44% included ethambutol and 13% included streptomycin as additional options. Although 71% of sites reported using efavirenz in the initial ART regimens in children older than three years of age during TB treatment, 79% used efavirenz or nevirapine.

### Antiretroviral drug access and monitoring

Sites reported having better access to stavudine-based (68% with easy access) than to zidovudine-based (25% with easy access) antiretroviral fixed-dose combinations for children. Atazanavir was not available or not used in children in 98% of African sites and 50% of Asian sites. There was no single dominant method to assess paediatric treatment adherence, with 40% of sites monitoring pill counts and 24% relying on RNA viral load testing. Multiple interventions to support adherence were utilized beyond counselling, including having a pharmacist on the core clinical team (73%), routine reviews of medication pick-up (67%) and pill boxes or blister packs (48%).

HIV DNA PCR, CD4 and RNA PCR testing were widely available across the regions, with reduced access to plasma HIV-1 RNA viral load in Central and West Africa (Table 2). CD4 was regularly monitored in 95% of sites, with less frequent monitoring of HIV-1 RNA (38%), creatinine (48%) and cholesterol levels (35%). Asian and Southern African sites had the highest overall levels of laboratory monitoring.

**Table 2:** Availability and utilization of diagnostic and monitoring testing*

	Asia	Central Africa	East Africa	Southern Africa	West Africa

Test	*N*=10	*N*=4	*N*=29	*N*=10	*N*=10
HIV DNA PCR					
- Available	9 (90)	3 (75)	29 (100)	10 (100)	5 (50)
- Turnaround time, median days (IQR)	14 (7–30)	30 (22–60)	30 (14–30)	9 (5–15)	14 (8.5–22)
CD4					
- Available	10 (100)	4 (100)	29 (100)	10 (100)	9 (90)
- Regularly monitored	10 (100)	3 (75)	28 (97)	10 (100)	9 (90)
- Turnaround time, median days (IQR)	3 (2–6)	3.5 (2.5–4.3)	14 (2.8–14)	3 (2–7)	5 (3–5)
HIV RNA PCR					
- Available	8 (80)	2 (50)	26 (90)	8 (80)	6 (60)
- Regularly monitored	9 (90)	2 (50)	1 (3)	7 (70)	5 (50)
- Turnaround time, median days (IQR)	10.5 (7–18)	21 and 30 days	30 (14–30)	7 (5–12)	26 (19–30)
ALT/AST					
- Available	10 (100)	4 (100)	29 (100)	10 (100)	9 (90)
- Regularly monitored	10 (100)	2 (50)	24 (83)	7 (70)	9 (90)
Creatinine					
- Available	10 (100)	4 (100)	29 (100)	10 (100)	9 (90)
- Regularly monitored	9 (90)	2 (50)	6 (21)	4 (40)	9 (90)
Total cholesterol					
- Available	10 (100)	4 (100)	29 (100)	9 (90)	8 (80)
- Regularly monitored	8 (80)	2 (50)	0	7 (70)	5 (50)
Tuberculosis-related**					
- Skin testing (e.g., purified protein derivative)	8 (80)	1 (25)	3 (10)	7 (70)	2 (20)
- Chest x-ray	10 (100)	3 (75)	28 (97)	9 (90)	6 (60)
- Acid-fast bacilli smear	10 (100)	3 (75)	14 (48)	10 (100)	5 (50)
- Acid-fast bacilli culture	8 (80)	1 (25)	3 (10)	8 (80)	3 (30)
- Drug susceptibility testing	7 (70)	0	0	9 (90)	2 (20)

IQR, interquartile range.

* Data listed as *N* (%) unless otherwise specified.

** Responses are not mutually exclusive.

## Discussion

The World Health Organization (WHO) and UNAIDS’ “Treatment 2.0” is a global framework whose objective is to achieve universal access for adults and children living with HIV infection through simplified treatment with optimized drug regimens, point-of-care diagnostics and decentralized service delivery [14]. Given that a major challenge for paediatric HIV care is to start all children under two years of age on ART, a critical step in achieving these goals is to better understand current programme resources and operations at the country and regional level.

Paediatric IeDEA is a multicentre, international collaboration of paediatric HIV treatment programmes in Asia and Africa that forms an operational research network to describe paediatric HIV care and treatment programmes, treatment outcomes and their determinants. Characterizing intra- and inter-country differences in current paediatric HIV care and treatment management programmes can help to guide the development of clinical practice guidelines, improve understanding of local resource availability and inform policy and programmes by examining various models of care, clinical outcomes and operational issues over time. However, ongoing changes inherent to progressive regional ART scale-up and international prevention and treatment guidelines make cross-regional comparisons over time even more challenging. In addition, the sites participating in this survey were predominantly in urban settings and the survey characterizes resources reflecting the highest levels of HIV care and treatment for children in these countries. Although they may not necessarily be representative of the local HIV programmes, many are national referral centres that provide advice and direction for paediatric HIV care throughout the different regions.

Although clinical resources and access to paediatric antiretroviral formulations were generally high, there was some variation between the regions. African and Asian sites notably differed with regards to infant feeding recommendations. International guidelines promote the use of exclusive breastfeeding due to higher levels of morbidity and mortality in non-breastfed infants [15,16], but some Asian countries continue to recommend exclusive formula feeding when feasible and provide support for free formula (e.g. Thailand, Vietnam, Malaysia, China) [17,18]. Some sites discontinued cotrimoxazole prophylaxis after immune recovery, despite the current WHO recommendations to continue it. With regards to antiretroviral drug availability, African sites tended to have easier access to tenofovir and abacavir than Asian sites (data not shown), which may reflect the more frequent eligibility for US PEPFAR pricing for drug procurement in sub-Saharan Africa. Definitions of loss to follow-up were inconsistent, which may impact future reporting. In addition, Central and West African sites tended to have less frequent viral load monitoring.

## Conclusions

To assess the long-term outcomes of ART in perinatally infected children as they age into adolescence, national paediatric HIV programmes in resource-constrained settings will need to expand their focus beyond diagnosis and early monitoring to a chronic disease model. More complex laboratory monitoring and targeted sub-cohort studies of toxicities and disease complications will also be needed to understand the impact of HIV and its treatment on adolescents and perinatally infected adults. Multiregional platforms like IeDEA have the capacity to facilitate this more advanced research agenda, promote data harmonization and guide national programmes and inform global policy.

## Writing Committee

*Asia-Pacific*: AH Sohn and A Kariminia, regional representatives; amfAR – The Foundation for AIDS Research, USA and Thailand; Kirby Institute, Australia; grant U01AI069907.

*Central Africa*: J Newman, regional representative; RTI International, USA; grant U01AI069927.

*East Africa*: K Wools-Kaloustian and S Ayaya, regional representatives; Indiana University, United States; grant U01AI69911.

*Southern Africa*: MA Davies and M Egger, regional representatives; University of Cape Town and the University of Bern, Switzerland; grants U01AI69924 and U01AI069924-05S1.

*West Africa*: V Leroy and D Ekouevi, regional representatives; Inserm U897, Institut de Santé Publique, d'Epidémiologie et de Développement, Université Bordeaux Segalen, Bordeaux, France; grants R01 AI058736 and U01AI09919.

*National Institutes of Health Pediatric Working Group Representatives:* M Bacon, National Institute of Allergy and Infectious Diseases; LM Mofenson, Eunice Kennedy Shriver National Institute of Child Health and Human Development.

## Appendix

**The TREAT Asia Pediatric HIV Network:** V Saphonn,* S Saramony, National Centre for HIV/AIDS Dermatology and STDs, Phnom Penh, Cambodia; U Vibol,* S Sophan, National Pediatric Hospital, Phnom Penh, Cambodia; J Tucker, New Hope for Cambodian Children, Phnom Penh, Cambodia; FJ Zhang, Beijing Ditan Hospital, Capital Medical University, Beijing, China; N Kumarasamy,* S Saghayam, YR Gaitonde, Centre for AIDS Research and Education, Chennai, India; N Kurniati,* D Muktiarti, Cipto Mangunkusumo, General Hospital, Jakarta, Indonesia; SM Fong,* M Thien, Hospital Likas, Kota Kinabalu, Malaysia; NK Nik Yusoff,* LC Hai, Hospital Raja Perempuan Zainab II, Kelantan, Malaysia; KA Razali,* Thahira JM, NF Abdul Rahman, Pediatric Institute, Hospital Kuala Lumpur, Kuala Lumpur, Malaysia; R Nallusamy,* KC Chan, Penang Hospital, Penang, Malaysia; V Sirisanthana,* L Aurpibul, Chiang Mai University, Chiang Mai, Thailand; R Hansudewechakul,* P Taeprasert, Chiangrai Prachanukroh Hospital, Chiang Rai, Thailand; P Lumbiganon,* P Kosalaraksa, P Tharnprisan, Khon Kaen University, Khon Kaen, Thailand; G Jourdain, Program for HIV Prevention and Treatment, Chiang Mai, Thailand; J Ananworanich,* C Phasomsap, T Suwanlerk, The HIV Netherlands, Australia, Thailand Research Collaboration (HIV-NAT), Bangkok, Thailand; K Chokephaibulkit,* W Phongsamart, O Wittawatmongkol, Siriraj Hospital, Mahidol University, Bangkok, Thailand; HK Truong,* DAN Mai, Children's Hospital 1, Ho Chi Minh City, Vietnam; CV Do,* MT Ha, Children's Hospital 2, Ho Chi Minh City, Vietnam; BV Huy,* VL Nguyen, National Hospital of Pediatrics, Hanoi, Vietnam; NO Le, Worldwide Orphans Foundation, Ho Chi Minh City, Vietnam; AH Sohn,* N Durier, J Pang, TREAT Asia, amfAR – The Foundation for AIDS Research, Bangkok, Thailand; DA Cooper, MG Law,* A Kariminia, The Kirby Institute, University of New South Wales, Sydney, Australia (*Steering Committee members).

**The IeDEA Central Africa Steering Group (2006–2011):** Dr. Théodore Niyongabo, Dr. Helene Bukuru, Dr. Martin Nduwimana and Dr. Pierre Kariyo of Centre Hospitalo-Universitaire de Kamenge, Bujumbura Burundi; Dr. Marcel Mbaya, Dr. Henri Mukumbi and Dr. Béatrice Ilunga of Amo-Congo, Democratic Republic of Congo; Dr. Marie-Thérèse Abena Obama and Dr. Nelly Kamgaing of Centre Hospitalier et Universitaire de Yaoundé, Yaoundé Cameroon; Dr. Wilfred Akam of Limbé Provincial Hospital, Limbé, Cameroon; IeDEA Central Africa Regional offices: Mr. Joseph Atibu, Mr. Innocent Azinyue, Dr. Modeste Kiumbu, Mr. Christian Kondé, Dr. John Ditekemena and Mr. Kashamuka Mwandagalirwa; and RTI International: Dr. Jamie Newman and Ms. Jennifer Hemingway-Foday.

**The IeDEA East Africa Steering Group (2006–2011):** We acknowledge the contributions of the East African IeDEA sites to this analysis and would like to thank Dr. Ayaya at the United States Agency for International Development – Academic Model Providing Access To Healthcare (USAID–AMPATH) Program, Eldoret, Kenya; Dr. Bukusi at Family AIDS Care and Education Services (Faces), Kisumu, Kenya; Dr. Lyamuya at Morogoro Regional Hospital, Tanzania; Dr. Maruchu at Tumbi Regional Hospital, Tanzania; Dr. Otieno at Nyanza Provincial Hospital, Kenya; Dr. Okong St. Francis Nsambya Hospital, Kampala, Uganda; Dr. Deo Wabwire, Makerere University, Kampala, Uganda; Dr. Masaba, Mbale Regional Hospital, Uganda; Drs. Elul and Nuwagaba-Biribonwoha at Columbia University's Mailman School of Public Health, USA and Dr. Cohen at University of California San Francisco, USA; Mr. E. Sang, Ms. E. Rotich and Ms. M. Achieng from the East African IeDEA Regional Data Center for their contributions.

**The IeDEA Southern Africa Steering Group**: Member Sites: *Cleophas Chimbetete, Newlands Clinic, Harare, Zimbabwe; *Brian Eley, Red Cross Children's Hospital, Cape Town, South Africa; Christiane Fritz, SolidarMed Zimbabwe, Zimbabwe; *Daniele Garone, Khayelitsha ART Programme and Médecins Sans Frontières, Cape Town, South Africa; *Janet Giddy, McCord Hospital, Durban, South Africa; Christopher Hoffmann, Aurum Institute for Health Research, South Africa; Patrick MacPhail, Themba Lethu Clinic, Helen Joseph Hospital, Johannesburg, South Africa; *Harry Moultrie, Wits Institute for Sexual Reproductive Health, HIV & Related Diseases, Faculty of Health Sciences, University of the Witwatersrand, Johannesburg, and Harriet Shezi Children's Clinic, Chris Hani Baragwanath Hospital, Soweto, South Africa; James Ndirangu, Hlabisa HIV Treatment and Care Programme, South Africa; Sabrina Pestilli, SolidarMed Mozambique, Mozambique; *Sam Phiri, Lighthouse Clinic, Lilongwe, Malawi; *Hans Prozesky and Helena Rabie, Tygerberg Academic Hospital, Stellenbosch, South Africa; Jeff Stringer, Center for Infectious Disease Research in Zambia, Zambia; *Karl Technau, Empilweni Service and Research Unit, Rahima Moosa Mother and Child Hospital, University of the Witwatersrand, Johannesburg, South Africa; *Paula Vaz, Paediatric Day Hospital, Maputo, Mozambique; *Robin Wood, Gugulethu and Masiphumelele ART Programmes and Desmond Tutu HIV Centre, Cape Town, South Africa. Central Team: Matthias Egger, Claire Graber, Fritz Kaeser, Olivia Keiser, Institute of Social and Preventive Medicine, University of Bern, Bern, Switzerland; Andrew Boulle, Morna Cornell, Mary-Ann Davies, Nicola Maxwell, School of Public Health and Family Medicine, University of Cape Town, Cape Town, South Africa.

*Sites that contributed data to this analysis.

**The IeDEA West Africa Working Group**: Primary Investigators: Pr François Dabis* (Inserm, U897, ISPED, University Bordeaux Segalen, Bordeaux, France), Pr Emmanuel Bissagnene* (SMIT, CHU de Treichville, Abidjan, Côte d'Ivoire). Clinical Investigators by country and alphabetical order (*Member of the IeDEA West Africa Technical Committee): Jocelyn Akakpo, Alain Azondékon, Jules Bashi, Sagbo Gratien, Sikiratou Koumakpaï, Marcel D. Zannou* (Benin); Ye Diarra, Eric-Arnaud Diendere, Joseph Drabo*, Fla Koueta (Burkina Faso); Edmond Aka-Addi, Clarisse Amani-Bosse, Franck-Olivier Ba-Gomis, François Eboua-Tanoh, Serge-Paul Eholie*, Calixte Guehi, Kouakou Kouadio, Serge-Olivier Koulé, Eugène Messou, Albert Minga, Aristophane Tanon, Marguerite Timité-Konan, Pety Touré, (Côte d'Ivoire); Kevin Peterson* (Gambia); Bamenla Goka, Lorna Renner* (Ghana); Hadizatou Coulibaly, Fatoumata Dicko, Moussa Maiga*, Daouda Minta, Mariam Sylla, Hamar Alassane Traoré; Man Charurat* (Man Charurat); Bernard Diop, Fatou Ly Ndiaye, Papa Salif Sow, Haby Signaté Sy*, Judicaël Tine (Senegal). Epidemiology and Statistical Unit (Inserm, U897, ISPED, Université Bordeaux Segalen, Bordeaux, France): Eric Balestre, Didier K. Ekouévi*, Antoine Jaquet*, Valériane Leroy*, Charlotte Lewden*, Karen Malateste, Annie Sasco, Rodolphe Thiebaut. Data Management Unit (PACCI, CHU Treichville, Abidjan, Côte d'Ivoire): Gérard Allou, Jean Claude Azani, Patrick Coffie. Paediatric clinical centres by city and country: Abidjan, Côte d'Ivoire: ACONDA-CEPREF, ACONDA-MTCT-Plus, CHU de Yopougon, Centre Intégré de Recherche Bioclinique d'Abidjan (CIRBA). Accra, Ghana: Korle Bu Teaching Hospital, Bamako. Mali: Hôpital Gabriel Touré. Cotonou. Benin: Centre National Hospitalo-Universitaire Hubert Maga, Hôpital d'Instruction des Armées. Dakar, Senegal: Hôpital d'Enfants Albert-Royer. Fajara, Gambia: Medical Research Council. Ouagadougou, Burkina-Faso: Centre Hospitalier Charles de Gaulle. Administration: Alexandra Doring and Elodie Rabourdin (ISPED), Hughes Djétouan, Bertin Kouadio and Adrienne Kouakou (PACCI).
